# Neuromyelitis optica spectrum disorder with active replication of hepatitis B virus and seropositive anti-aquaporin-4 antibody: A case report

**DOI:** 10.1097/MD.0000000000027207

**Published:** 2021-09-24

**Authors:** Jiaying Lei, Hong Wang

**Affiliations:** Qilu Hospital of ShanDong University, Jinan, China.

**Keywords:** aquaporin-4, corticosteroid, HBV, liver function, neuromyelitis optica spectrum disorder

## Abstract

**Rationale:**

Neuromyelitis optica spectrum disorder (NMOSD) associated with active replication of hepatitis B virus (HBV) is rare. High-dose corticosteroids are the mainstay treatment of NMOSD; however, these may cause reactivation of viral replication in patients with stable HBV which may lead to liver damage. Therefore, care should be placed in corticosteroid use in patients with NMOSD and HBV infection.

**Patient concerns:**

Herein, we report the case of a 31-year-old woman with NMOSD and HBV infection who was seropositive for anti-aquaporin-4 antibody. The stable and HBV carrier status of the patient led to the deferment of antiviral and hepatoprotective agents in early treatment. However, liver function impairment was detected during follow-up, with an improvement in the best-corrected visual acuity.

**Diagnoses:**

The patient was diagnosed with NMOSD with active replication of HBV and seropositive anti-aquaporin-4 antibody considering the medical history and ancillary examinations.

**Interventions:**

To manage NMOSD, intravenous high-dose methylprednisolone (20 mg/kg d) was administered for 5 days which was gradually tapered to oral steroids. However, liver function impairment was observed during follow-up; therefore, anti-HBV drugs (entecavir) and hepatoprotective drugs (bicyclol or polyunsaturated phosphatidylcholine) were administered.

**Outcomes:**

A marked improvement was observed in the patient's best-corrected visual acuity after 4 weeks of treatment. However, follow-up examinations revealed liver function damage which necessitated administration of antiviral and hepatoprotective drugs. Liver function normalized after 1 month.

**Lesson:**

This case underscores the importance of preventive treatment of liver protection in patients with HBV infection prior to or simultaneous with glucocorticoid therapy and furthermore, there is an urgent need to develop authoritative guidelines regulating corticosteroid use in the treatment of patients with HBV infection.

## 1 Introduction

Neuromyelitis optica (NMO) is an acute or subacute demyelinating inflammatory disease of the central nervous system (CNS), traditionally understood to be confined to the optic nerve and spinal cord. However, there is a group of demyelinating diseases with a limited form that do not meet the clinical diagnostic criteria for NMO. Similar pathogenesis and clinical characteristics are observed between this group and NMO, including single or recurrent optic neuritis (ON) and longitudendelitis extensive transverse myelitis, ON or longitudendelitis extensive transverse myelitis with rheumatoid immune disease, and positive rheumatoid immune-related autoimmune antibodies. In 2007, Wingerchuk et al^[1]^ named this group of diseases as neuromyelitis optica spectrum disorder (NMOSD). However, previous research has found no significant difference regarding the biological characteristics and treatment strategies between NMO and NMOSD; furthermore, patients with NMOSD eventually develop NMO. In June 2015, the International Panel for NMO Diagnosis revised the nomenclature and diagnostic criteria for NMO; particularly, they removed the separate definition of NMO and included NMO in the broader category of NMOSD.^[2]^

Chronic hepatitis B (CHB) infection is a global health problem. In 2009, more than 2 billion people have been infected with hepatitis B virus (HBV) worldwide.^[3]^ In China, the infection rate of HBV is higher, and approximately 20% of patients with HBV infection exhibit extrahepatic manifestations.^[4]^ HBV infection and vaccination are associated with CNS demyelinating diseases, such as ON, transverse myelitis, acute disseminated encephalomyelitis, and multiple sclerosis.^[5–8]^ Furthermore, previous studies in the US have investigated the incidence of NMOSD postHBV vaccination.^[9]^ Zhao et al^[7]^ found that patients with NMOSD and concomitant CHB infection exhibited severe manifestations. Liu et al^[5]^ analyzed the clinical features of 10 Chinese patients with NMOSD combined with CHB infection who were seropositive for aquaporin-4; they explained the possible pathologic mechanism between the association of NMOSD and HBV infection. However, the role of HBV infection in the emergence of NMOSD was not specified, and no recommendations regarding the use of corticosteroids in patients with NMOSD and HBV infection were outlined.

Here, we report a case of NMOSD with CHB infection who was seropositive for aquaporin-4-antibodies.

## 2 Case presentation

### 2.1 Patient information

**Figure 1:**
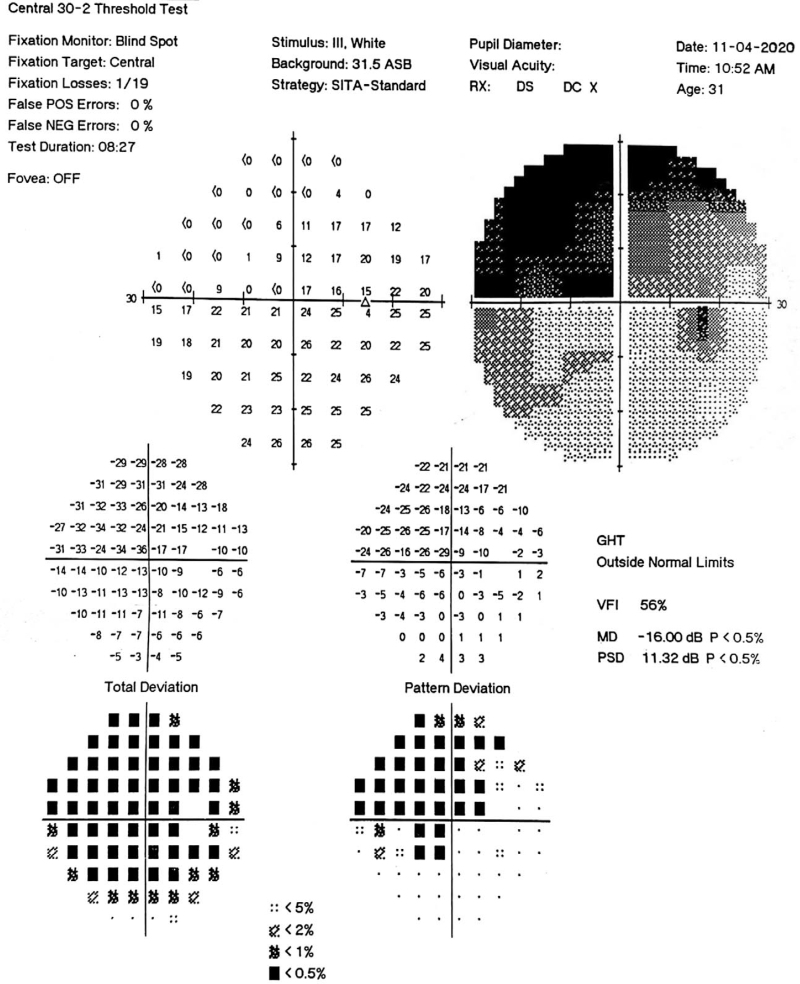
Visual field examination of right eye upon admission showed a defect of upper visual field in the right eye.

In November 14, 2020, a 31-year-old woman consulted our hospital due to acute vision loss of the right eye for 1 week. This was associated with lightening of the visual object and painful eye rotation for 1 week. One week prior to admission, the visual color above the nose of the right eye turned white; however, no triggering factors were identified. Upon admission, visual field examination was performed which revealed “partial visual defect of the right eye” (Fig. 1). She experienced rapid decrease in her right eye visual acuity, which progressed to “hand motion” 1 day before admission. One year prior to consultation, she was diagnosed as an asymptomatic HBV carrier and received no treatment. However, further probing of past medical history was unremarkable for other comorbid diseases. She denies history of trauma and surgery. Furthermore, she is allergic to mango and ultraviolet light. She had a regular routine, and denies history of tobacco and alcohol use. Her family history was unremarkable.

### 2.2 Clinical findings

After admission, the visual acuity of the right eye was hand motion (–11.00/–2.50∗150 = no improvement), and the left eye was 0.08 (–14.00/–3.50∗175 = 0.8). The intra-ocular pressure of both eyes were 19 mm Hg. The pupil diameter of the right and left eye were approximately 5 and 3 mm, respectively. Additionally, the penlight examination of the right eye revealed absence of the direct light pupillary reflex, presence of the indirect light pupillary reflex, and positive RAPD; on the other hand, the indirect and direct light pupillary reflex of the right eye were both present. Upon fundoscopic examination, the optic disc boundary of both eyes was clear with a light red color; furthermore, no reflection was observed in the fovea of the macula.

### 2.3 Diagnostic assessment

The flash visual evoked potential showed a delay in the P2 peak time and decrease in the P2 wave amplitude of the right eye compared with the left eye. Results from the optical disc optical coherence tomography, electrocardiogram, chest radiography, spinal cord magnetic resonance imaging (MRI), routine complete blood count, urinalysis, coagulation studies, erythrocyte sedimentation rate, C-reactive protein, anti-nuclear antibody spectrum 17 items, treponema pallidum specific antibody, and liver and kidney function tests were unremarkable. Furthermore, HBV profile revealed (+) HBsAg, (+) HBeAg (+), (–) HBeAb, (+) HBcAb-IgG, (–) HCV-Ab, (–) HCV-Ag, and (+) PreS1-Ag. Antibodies against demyelinating diseases of the CNS revealed seropositive anti-aquaporin-4 antibody (AQP4-Ab). Computer visual field: defect of the upper visual field of the right eye (Fig. 1). MRI scans of the brain showed a long T2 signal in the intra-orbital segment of the right optic nerve, consistent with ON. The patient was diagnosed with the following conditions considering the medical history, and physical and ancillary examinations:

1. NMOSD,
2. binocular ametropia,
3. stage of active HBV replication.

### 2.4 Therapeutic intervention

During the first week, intravenous high-dose methylprednisolone (20 mg/kg d) was administered for 5 days with drugs that nourish the nerve and reduce adverse hormone effects; the methylprednisolone were tapered to oral prednisone tablets therapy (1 mg/kg d) after 5days. She remained in the hospital for 10 days. Prior to discharge, improvements were observed in the patient's visual acuity, color vision, and right eye shapes and details. Particularly, the visual acuity of the right eye was “finger count”/30 cm (–11.00/–2.50∗150 = no improvement), and the left was 0.08 (–14.00/3.50∗175 = 4.9).

### 2.5 Follow-up and outcomes

**Figure 2:**
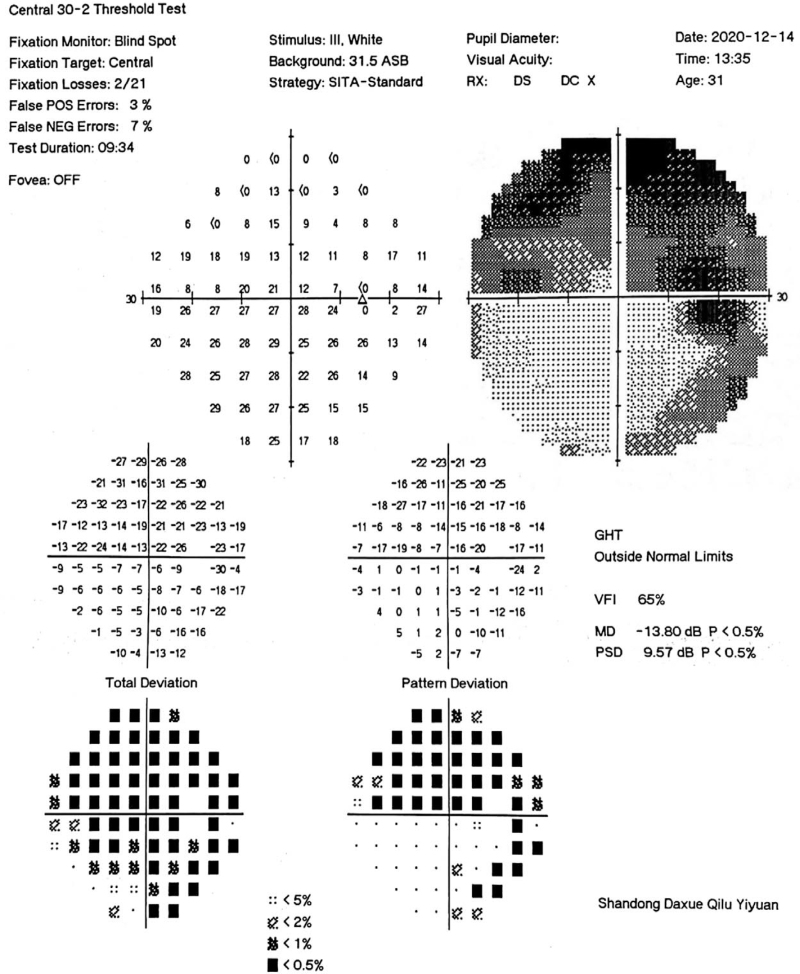
Visual field examination of right eye at first review showed that the area of visual field defect in the right eye was slightly improved compared with that at admission.

Following discharge, the patient was maintained on oral prednisone tablets therapy whose dosage was gradually reduced by 5 mg every 2 weeks. This was administered with adjuvant drugs to nourish the nerve and reduce adverse hormone reactions. On December 14, 2020, patient was followed up. During this consult, visual examination revealed best-corrected visual acuity of the right and left eyes as 0.1 and 0.8, respectively. A visual field examination is shown in Figure 2. Her second follow-up was on January 4, 2021 which revealed best-corrected visual acuity of the right and left eye as 0.12 and 0.8, respectively.

On February 21, 2021, the patient felt limited visual field and unsatisfactory improvement of the right eye; therefore, she was admitted in the General Hospital of the People's Liberation Army for 7 days. On admission, the visual acuity of right eye was 0.04 (–14.5/–2.50∗180 = 0.6), while that of the left eye was 0.08 (–14.50/2.50∗175 = 0.8). Additionally, upon analysis, cerebrospinal fluid and blood were reactive for HBsAg, HBeAg, and core antibodies; furthermore, quantitative determination of HBV DNA was 8.44 × 10^7^ copies/mL. T-cell analysis was performed for tuberculosis infection, which revealed antigen A (–), antigen B (–), anti-cardiolipin antibodies (–), and HLA-B27(–). Furthermore, liver function tests revealed that alanine aminotransferase and aspartate aminotransferase were 287.5 U/L and 272.1 U/L, respectively. The patient received a reduced dose of oral prednisone tablets therapy (20 mg/d) combined with adjuvant drugs to improve microcirculation, nourish nerve, anti-HBV drugs (entecavir 0.5 mg/d), and hepatoprotective drugs (bicyclol 25 mg tid, or polyunsaturated phosphatidylcholine 228 mg tid). Once the patient's condition was stable, she was discharged. On March 22, 2021, 1 month after discharge, her liver function tests and HBV DNA quantification were re-evaluated. The results revealed alanine aminotransferase and aspartate aminotransferase of 20.4 U/L and 80.3 U/L respectively, indicating a return to normal for the patient's liver function. Quantitative determination of HBV DNA revealed 1.46 × 10^3^ copies/mL, indicating a significant decrease in the HBV replication activity.

## 3 Discussion

NMOSD is a group of inflammatory demyelinating diseases mediated by antigens and antibodies of the humoral immunity which affects the CNS. AQP4 is a protein located in the cell membrane of neurons, such as astrocytes. The international consensus for NMOSD diagnosis revised in 2015 proposed to divide NMOSD into NMOSD with or without AQP4-IgG.^[2]^ For patients with AQP4-IgG, only 1 of the following core clinical features is required to be diagnosed with NMOSD. These core clinical features include clinical syndromes or MRI findings related to spinal cord, optic nerve, area postrema, other brainstem, cerebral, or diencephalic presentations. The detection of AQP4-Ab is 99% specific to NMO disease; a seropositive AQP4-Ab is needed to establish a diagnosis of NMO or NMOSD, which was observed in our patient. ON is an inflammatory lesion of the optic nerve caused by various causes,^[10]^ According to etiology, it can be classified as idiopathic, autoimmune, infectious, or unclassified ON. Notably, precursors of viral infections may induce factors causing idiopathic ON. Furthermore, infection-related ON can lead to autoimmune disorders when the infection triggers whole-body or local inflammatory response of the optic nerve; therefore, overlaps may be observed between idiopathic and infectious ON in some classifications. In 2014, Toosy et al^[10]^ proposed the concept of typical and atypical ON according to the relationship of ON to either multiple sclerosis or demyelinating disease of the CNS. Zhao et al^[7]^ showed that 92.3% of patients with ON and HBV infection were atypical. Our patient reported no history of autoimmune diseases and infection or close contact, except for being diagnosed as an HBV carrier. Additionally, anti-nuclear antibody spectrum test results were unremarkable. Therefore, differential diagnosis between infectious ON and connective tissue disease-induced optic neuropathy. The patient lived their life routinely and reported no history of smoking, alcohol drinking, or gastrointestinal disease; these factors exclude the diagnosis of toxic and malnourished metabolic optic neuropathy. The brain MRI findings results suggest that only the right optic nerve signal was abnormal; therefore, visual acuity loss caused by compression of intra-cranial tumor or sinus inflammation involving the optic nerve could be ruled out. In addition, the young age of the patient, and the presentation of decreased vision with ocular rotation pain and unremarkable fundoscopic examination rule out Leber hereditary optic neuropathy. Therefore, it was considered that the disease was caused by HBV infection, resulting in autoimmune dysfunction which caused ON symptoms.

It has been reported that HBV infection may aggravate ON.^[7]^ Furthermore, a confirmation of a patient's HBV infection or vaccination status and the presence of clinical manifestations that meet the NMOSD diagnostic criteria are required to establish a diagnosis of HBV-associated NMOSD. In our patient, HBeAg was positive, and quantitative determination of HBV DNA was 8.44 × 10^7^ copies/mL, indicating active HBV replication. Furthermore, her AQP4-Ab IgG level was serologically positive. Therefore, the diagnosis of HBV-associated NMOSD was confirmed after exclusion of other infectious factors. Liu et al^[5]^ speculated that the molecular simulation and immune cross-reaction between HBsAg and myelin antigen of the CNS may potentially explain the primary pathophysiologic mechanisms underlying NMOSD with HBV infection. Molecular simulation of T cell infective factors has been suggested as a potential mechanism of demyelination of the CNS after HBV vaccination.

At treatment initiation, the patient was only an HBV carrier with normal liver functions; therefore, antiviral and hepatoprotective medications were not administered; however, continuous glucocorticoid administration for 2 months led to liver damage. To address this, the steroid dose was gradually tapered while administering antiviral and hepatoprotective medications. After 1 month, liver function gradually returned to normal and HBV replication was controlled. An increased risk of hepatitis flare is observed in patients with CHB undergoing glucocorticoid therapy even if administered as short-term and high dose; therefore, researchers suggest the effectiveness of prophylactic antiviral therapy in patients receiving high-dose glucocorticoids regardless of duration.^[11]^ Curras-Martin et al^[12]^ reported a 46-year-old African-American man with a history of untreated HBV infection, who presented with blurred vision; he was diagnosed with ON with chronic or acute HB infection, twice. The first treatment was a combination of hormonal and antiviral therapy. The second treatment was considered to be viral hepatitis flare; therefore, steroids were avoided and only antiviral medication was administered. In both treatments, the patient's visual acuity improved after a few days; furthermore, in the second treatment, after 5 days of treatment with antiviral drugs, a rapid recovery of the liver function and gradual reduction of symptoms of systemic discomfort were observed. Liu et al.^[5]^ retrospectively analyzed 10 patients with NMOSD with CHB infection who were seropositive for AQP4-Ab; they found an increased risk of liver damage in these patients after high-dose glucocorticoid therapy. Therefore, they suggested that patients with NMOSD should be aware of the possibility of HBV infection once liver damage occurs, which would necessitate adjustments to treatment. Teng et al^[13]^ reported a case of hepatitis B-related optic neuritis that was treated with therapeutic plasma exchange. This finding demonstrated the correlation between hepatitis B-related optic neuritis and immunopathogenesis, which presents an alternative treatment for hormone-invalid and disabled ON.

However, a standardized guideline of treatment of patients with HBV and NMOSD remains uncreated. Furthermore, conflicts regarding the medication remain for patients with hepatitis B complicated with NMOSD. These conflicts are attributed to the liver damage caused by high-dose glucocorticoids in patients with HBV despite it being the routine treatment of patients with NMOSD. This case report suggests the importance of glucocorticoid therapy with antiviral drugs in the management of asymptomatic HBV carriers.

## Author contributions

Jiaying Lei presented the idea, learned about optic neuritis, hepatitis B virus, and immune complex disease, she also wrote the manuscript. Hong Wang reviewed and corrected the manuscript.

**Conceptualization:** Hong Wang.

**Formal analysis:** Jiaying Lei.

**Investigation:** Jiaying Lei.

**Supervision:** Jiaying Lei.

**Writing – original draft:** Jiaying Lei.

**Writing – review & editing:** Hong Wang.

## References

[1] Wingerchuk DM Lennon VA Lucchinetti CF Pittock SJ Weinshenker BG. The spectrum of neuromyelitis optica. Lancet Neurol 2007;6:805–15. 17706564 10.1016/S1474-4422(07)70216-8

[2] Wingerchuk DM Banwell B Bennett JL. International consensus diagnostic criteria for neuromyelitis optica spectrum disorders. Neurology 2015;85:177–89. 26092914 10.1212/WNL.0000000000001729 PMC4515040

[3] Hepatitis B vaccines. WHO position paper. Wkly Epidemiol Rec 2009;84:405–19.19817017

[4] Cacoub P Terrier B. Hepatitis B-related autoimmune manifestations. Rheum Dis Clin North Am 2009;35:125–37. 19481001 10.1016/j.rdc.2009.03.006

[5] Liu J Xu L Chen ZL Li M Yi H Peng FH. Comprehensive analysis of patients with neuromyelitis optica spectrum disorder (NMOSD) combined with chronic hepatitis B (CHB) infection and seropositive for anti-aquaporin-4 antibody. Bosn J Basic Med Sci 2018;18:35–42. 29144890 10.17305/bjbms.2017.2255 PMC5826672

[6] Lazibat I Brinar V. Acute disseminated encephalomyelitis associated with hepatitis B virus reinfection—consequence or coincidence? Clin Neurol Neurosurg 2013;115: (Suppl 1): S35–7. 24321152 10.1016/j.clineuro.2013.09.018 PMC7116977

[7] Zhao S Chen T Peng C. The putative acceleration of optic neuritis when combined with chronic hepatitis B. J Neurol Sci 2015;358:207–12. 26363926 10.1016/j.jns.2015.08.1538

[8] Jiang ZC Liu ZH Li HY Wei SH. Optic neuritis combined with hepatitis B and neurosyphilis. Chin Med J (Engl) 2013;126:3580–1. 24034115

[9] Geier DA Geier MR Geier MR. A case-control study of serious autoimmune adverse events following hepatitis B immunization. Autoimmunity 2005;38:295–301. 16206512 10.1080/08916930500144484

[10] Toosy AT Mason DF Miller DH. Optic neuritis. Lancet Neurol 2014;13:83–99. 24331795 10.1016/S1474-4422(13)70259-X

[11] Wong GL Yuen BW Chan HL. Impact of dose and duration of corticosteroid on the risk of hepatitis flare in patients with chronic hepatitis B. Liver Int 2019;39:271–9. 30179316 10.1111/liv.13953

[12] Curras-Martin D Campbell N Haroon A Hossain MA Asif A. Recurrent optic neuritis as the only manifestation of chronic hepatitis B virus flare: a case report. BioMed Central 2018;1:12. 10.1186/s13256-018-1810-0 PMC6192156 30326966

[13] Teng D Tan S Yang M. A case report of hepatitis B related optic neuritis treated with plasma exchange. Medicine 2019;98:e15432. 31045806 10.1097/MD.0000000000015432 PMC6504246

